# 1,9-Dimethyl-methylene Blue-Based Antimicrobial Photodynamic Inactivation: Sulfate-Reducing Bacteria Models Isolated from Oilfield Wastewater

**DOI:** 10.1021/acsomega.5c09000

**Published:** 2026-04-20

**Authors:** Hesrom Fernandes Serra Moura, Igor Carvalho Fontes Sampaio, Gustavo Vital dos Santos, Anna Paula Lima Teixeira da Silva, Pedro Jorge Louro Crugeira, Wellington Luis Reis Costa, Antonio Luiz Barbosa Pinheiro, Paulo Fernando de Almeida

**Affiliations:** † Center of Biophotonics, School of Dentistry, 495455 Federal University of Bahia − UFBA, 62 Araújo Pinho Ave, Canela, Salvador, Bahia CEP: 40110−150, Brazil; ‡ Biotransformation and Organic Biocatalysis Research Group, Department of Exact Sciences, Universidade Estadual de Santa Cruz, 45654-370 Ilhéus, Brazil; § CIMO, LA SusTEC, 70863 Instituto Politécnico de Bragança, Campus de Santa Apolónia, 5300-253 Bragança, Portugal; ⊥ Laboratory of Biotechnology and Ecology of Microorganisms, Institute of Health Sciences, 28111 Federal University of Bahia − UFBA, Av. Reitor Miguel Calmon, S/N, 40110-100 Salvador, Bahia, Brazil

## Abstract

Sulfate-reducing bacteria (SRB) are responsible for biogenic acidification, which negatively impacts oil quality and presents significant economic and environmental challenges for the oil industry. The improper use of biocides often leads to the emergence of biocide-resistant SRB strains, posing additional risks of contaminating aquatic environments. This study aimed to evaluate antimicrobial photodynamic inactivation (API) as a control strategy for a consortium of biocide-resistant sulfate-reducing bacteria collected from an oil extraction field in the Recôncavo Baiano Basin, Brazil, under conditions representative of oilfield microbial communities. 1,9-Dimethyl-methylene blue (DMMB) was used as a photosensitizer at concentrations of 1.0, 1.5, and 2.0 μg/mL, activated by LED light (λ 630 ± 10 nm, CW—Continuous wave, 125 mW) with energy densities of 8.0, 10.0, 12.0, 14.4, and 21.6 J/cm^2^. API using LED reduced bacterial log counts by up to 72.86% (0.57 log) after 9 min of treatment. The antimicrobial effects are consistent with a Type I API mechanism, involving the generation of reactive oxygen species (ROS) and hydroxyl radicals. The incomplete growth inhibition may be explained by complex interactions between DMMB and the lipopolysaccharide layers present in SRB of the genus *Desulfovibrio*. Additionally, H_2_S generated by SRB acted as a quencher, limiting the activity of singlet oxygen. These findings emphasize the intricate relationship between the choice of photosensitizer, the photoactivation conditions, and the presence of quenchers. They also contribute to understanding API performance in complex anaerobic microbial consortia and highlight its potential application for SRB control in oilfield environments.

## Graphical abstract

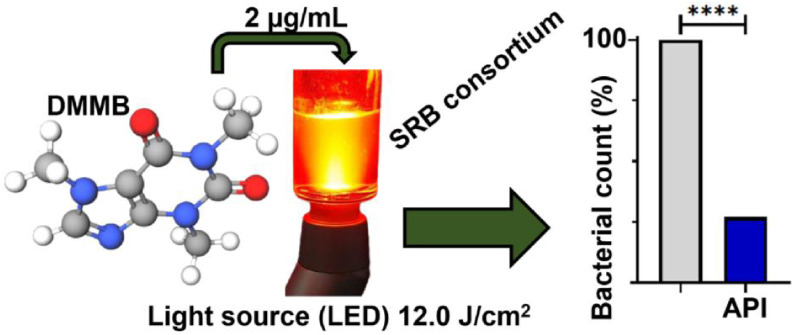

## 1 Introduction

Sulfate-reducing bacteria (SRB) are anaerobic microorganisms that thrive in temperatures between 25 and 44 °C and pH levels ranging from 5.5 to 9.0. These microorganisms are considered key agents in microbially influenced corrosion (MIC) of metals in marine environments.^1^ SRB utilize SO_4_^2–^ ions as terminal electron acceptors during anaerobic respiration. Under anaerobic conditions, SRB convert SO_4_^2–^ to sulfide (S^2–^ ), which combines with H^+^ in the environment to form the highly corrosive and toxic compound H_2_S. In the absence of sulfate, SRB can use other electron acceptors or different substrates, including certain hydrocarbons like phenols.^2^

Many oil reservoirs are anaerobic environments, favorable for the growth and metabolism of SRB, making them significant contributors to economic losses in the oil industry.^2^ SRB can grow and form biofilms on environmental contaminants such as petroleum hydrocarbons, including benzene, toluene, ethylbenzene, xylenes, naphthalene, phenanthrene, alkanes, and halogenated compounds.^3–5^

The formation of these biofilms leads to the development of corrosion on the metallic structures of pipelines and equipment, contamination of injection wells, bioaccumulation impacts on production rates and flow rates, and biogenic acidification caused by sulfide production in the form of hydrogen sulfide (H_2_S).^6^ To prevent these issues during oil extraction, a biocide treatment is commonly used. Some of the biocides employed include glutaraldehyde, dibromonitrilopropionamide, tetrakis­(hydroxymethyl)­phosphonium sulfate, and alkyl dimethyl benzyl ammonium chloride.^6,7^

One of the most critical challenges in using antimicrobial compounds is the development of resistance among SRB.^6^ Prolonged exposure to antimicrobial compounds can lead to the selection of resistant microorganisms, rendering standard treatments less effective.^8^ This resistance can arise from genetic mutations or horizontal gene transfer.^9^ Resistant bacteria can share their defense mechanisms with others, making it necessary to use higher concentrations or develop alternative biocides, which result in increased operational costs and environmental risks.^4,10^

The application of antimicrobial compounds in oil fields can have detrimental effects on the surrounding environment. These chemicals can persist, leading to soil and water contamination. The toxicity of certain biocides poses risks to nontarget organisms, including other microorganisms in aquatic environments and wildlife.^11^

Environmental concerns are increasingly being discussed in public policies, potentially limiting the types and quantities of harmful antimicrobial compounds that can be used, further complicating their application in oilfield settings. For these reasons, new microbial control approaches are being investigated.^5,11^

Antimicrobial photodynamic inactivation (API) techniques involve the use of a photosensitizer (usually a light-sensitive dye), which, after illumination with a light source (e.g., LED) of an appropriate wavelength, becomes activated. In this energized state, it can react with molecular oxygen present within and around cells to produce reactive oxygen species (ROS), including singlet oxygen through energy transfer or hydroxyl radicals through electron transfer, which are highly damaging to the target cell.^12^

Since API attacks multiple targets within bacteria, rather than a single target like biocides, the probability of developing antimicrobial resistance is reduced.^13–17^ Considering the negative impacts caused by SRB in the oil industry and the adverse consequences of biocide use, it is essential to develop an effective and safe bactericidal treatment. This study hypothesizes that an LED-based API can effectively inactivate SRB consortia isolated from real-world oil wells in a dose-dependent manner.

Among the available classes of photosensitizers for API, phenothiazines are hydrophobic, photosensitive compounds with a strong tendency to generate ROS. Their efficacy is determined by the photophysical and photochemical properties of their monomers. Their lipophilic nature is crucial for intracellular incorporation of the photosensitizer^18^ and contributes to a higher quantum yield of singlet oxygen^19^ as well as greater phototoxicity compared to other photosensitizers.^19^ 1,9-Dimethyl-Methylene Blue (DMMB), a phenothiazine derivative, has demonstrated promising results in API against diverse types of microorganisms.^20–22^ DMMB’s API is driven by its phenothiazinium chromophore, which enables efficient light absorption and generation of ROS.^23^ Upon illumination, DMMB forms a triplet state that transfers energy to molecular oxygen, producing singlet oxygen, a powerful cytotoxic agent.^24^

In this context, this study evaluated the in vitro effects of API using DMMB as a photosensitizer at different concentrations (0.050, 0.250, 0.500, 0.750, 1.0, 1.5, and 2.0 μg/mL) combined with LED light at different energy densities (8.0, 10.0, 12.0, 14.4, and 21.6 J/cm^2^) on an SRB consortium isolated from produced water of the Alvorada and Camboatá oil wells, located in an oil extraction field in the Recôncavo Baiano Basin (Brazil). Although API using DMMB has previously been investigated in the same SRB consortium under laser irradiation, the present study advances this approach by evaluating LED-based activation as a technologically and operationally viable alternative. Unlike laser systems, LEDs offer lower acquisition and maintenance costs, broader irradiation coverage, improved safety, and greater scalability for industrial implementation. By maintaining comparable microbiological and physicochemical conditions, including anaerobic growth and active H_2_S production, this work enables a direct assessment of whether LED irradiation can achieve antimicrobial performance similar to or superior to laser-based activation while improving practical feasibility under conditions relevant to oilfield environments. Thus, the novelty of this study lies not in the use of DMMB itself, but in the translational evaluation of LED-driven API as a more accessible, scalable, and field-compatible strategy for controlling biocide-resistant SRB consortia.

## 2 Materials and Methods

### 2.1 Microbial Consortium

The microbial consortium used in this study is part of the microbiota present in produced water collected from the Alvorada and Camboatá oil wells, located in the Recôncavo Baiano Basin (Brazil). This consortium was previously identified by Rosário^25^ and provided by the Laboratory of Biotechnology and Ecology of Microorganisms (LABEM) at the Federal University of Bahia, Brazil. The consortium includes two strains of *Desulfovibrio vulgaris*; two strains of *Desulfovibrio alaskensis*; two strains of *Desulfovibrio dechloracetivorans*; two strains of *Desulfovibrio* sp.; one strain of *Desulfovibrio vulgaris* ATCC 29579; and one strain of *Thermovirga lienii*.^25^

### 2.2 Growth of SRB Consortium

The cryopreserved consortium was thawed at room temperature within an anaerobic chamber (Bactron VI, Shellab, Sheldon Manufacturing Inc.). After thawing, the consortium was inoculated into modified Postgate C medium (pH 7.5) contained in 10 mL penicillin bottles, which were purged with N_2_, sealed with rubber caps, and properly secured to prevent oxygen exposure.^10^ The bottles were then incubated at 37 °C for 12 h in a TE-391/1 bacteriological incubator (Tecnal, Piracicaba, Brazil) without agitation. Modified Postgate C medium composition is as follows: NaCl—1.0%/L; KH_2_PO_4_—0.05%/L; NH_4_Cl—0.1%/L; Na_2_SO_4_—0.1%/L; CaCl_2_—0.1%/L; MgCl_2_·6H_2_O—0.183%/L; yeast extract—0.1%/L; ascorbic acid—0.01%/L; sodium thioglycolate—0.0013%/L; sodium citrate—0.638%/L; sodium lactate—0.175%/L; resazurin—0.025%/L (m/v)—0.4%; FeSO_4_·7H_2_O—0.05%.^22^ Subsequently, a 10% (v/v) transfer was made to new 10 mL penicillin bottles containing 9 mL of fresh modified Postgate C medium and incubated in a bacteriological B.O.D. incubator (TE-391/1 TECNAL, Brazil) for 30 h at 37 °C without agitation. The experimental microbial culture had a concentration of 3.2 × 10^8^ cells/mL in the exponential growth phase, as there is a consensus in the literature that bacteria in exponential growth are more susceptible to antimicrobial agents.

### 2.3 DMMB Toxicity Assessment

The photosensitizer DMMB, a zinc chloride double salt of 1,9-dimethyl-methylene blue (Figure 1A, Sigma-Aldrich, St. Louis, MO, USA), with a MM of 347.905 g/mol^26^ and an absorption maximum (λmax) between 550 and 649 nm (Figure 1B), was used. The manufacturer reports a purity of approximately 80%, with the remaining fraction corresponding to bound water.

**Figure 1:**
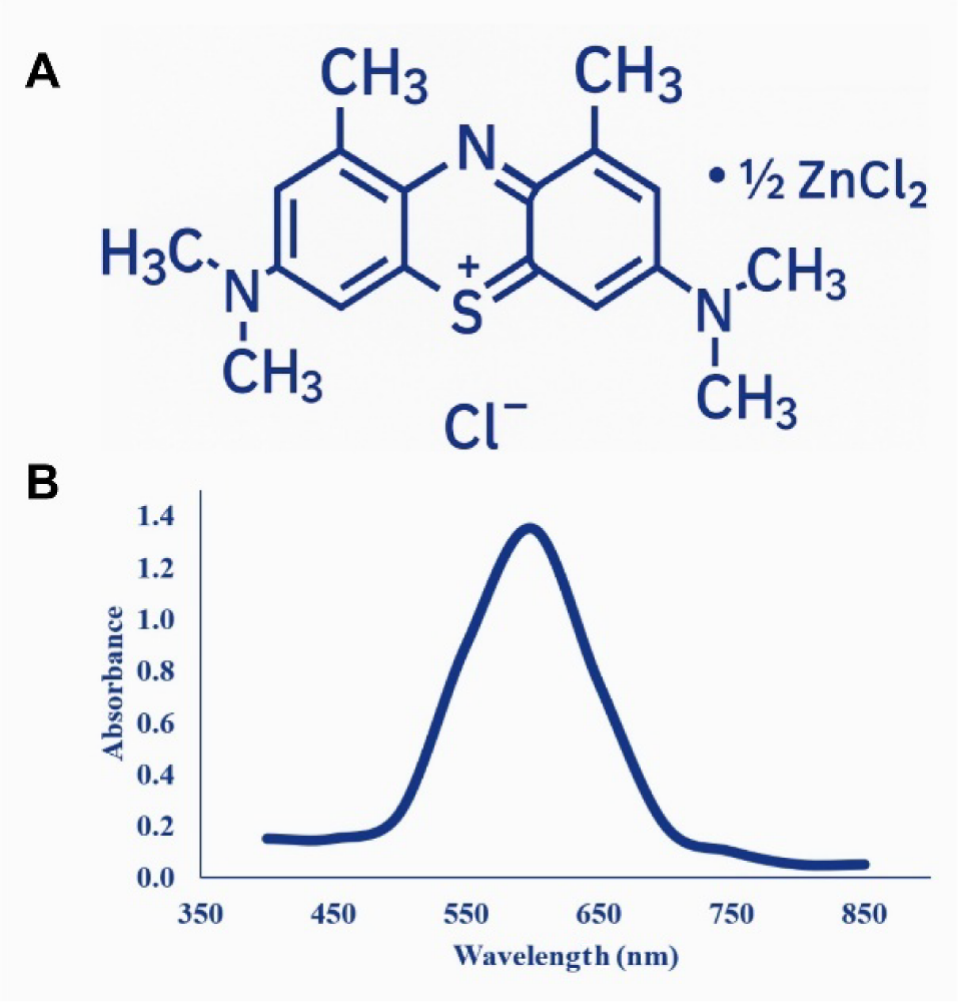
1,9-Dimethyl-Methylene Blue Zinc Chloride Double Salt (A) molecular structure. (B) UV–Vis absorption spectrum. Adapted from Journal of Photochemistry and Photobiology B: Biology, 200, Santos et al.,^27^ A novel technique of antimicrobial photodynamic therapy – aPDT using 1,9-dimethyl-methylene blue zinc chloride double salt-DMMB and polarized light on *Staphylococcus aureus*, 111646, Copyright 2019, with permission from Elsevier.

The mass of DMMB was measured on an analytical balance, then diluted in distilled water and filtered using a syringe and a sterile polyethersulfone filter (diameter 25 mm, pore 0.45 μm, Kasvi). Prior to DMMB treatment, each penicillin flask containing the experimental microbial consortium (Section 2.2) was covered with aluminum foil to protect it from light. To assess toxicity, each flask was treated with precise amounts of a concentrated DMMB solution to achieve final concentrations of 0.050, 0.250, 0.500, 0.750, 1.0, 1.5, and 2.0 μg/mL inside the anaerobic chamber. The control group and each experimental group were incubated for 5 min at 37 °C. After the exposure, 100 μL aliquots of the suspension were analyzed using a SpectraMax 190 spectrophotometer (Molecular Devices, California, USA) at λ 600 nm to determine the cell concentration, based on a calibration curve. Optical density at λ 600 nm (OD_600nm_) was used as a rapid and reproducible proxy for suspended particulate biomass. OD_600nm_ primarily reflects light scattering by bacterial-sized particles and therefore tracks relative changes in cell/biomass concentration across treatments; it does not directly measure metabolic viability. OD_600nm_ values were converted to estimated cells per milliliters using a proportional relationship based on McFarland turbidity standards.

### 2.4 Evaluation of Different Energy Densities

To increase the illuminated surface area, the collimating tip of the LED device was removed prior to the experiments. This tip normally concentrates the emitted light into a smaller spot; its removal allowed for greater beam divergence and a broader light distribution over the experimental surface. To prevent light dispersion beyond the region of interest, the experimental flasks were wrapped in aluminum foil, leaving only the bottom surface exposed to the irradiation.

The light source used was an LED (Fisioled, MMOptics, São Carlos, SP, Brazil, λ 630 ± 20 nm, 125 ± 5 mW, CW (Continuous wave)) with energy densities of 8.0, 10.0, 12.0, 14.4, and 21.6 J/cm^2^. The equipment was calibrated before the experimental procedures using a photometer (Thorlabs PM30, Newton, NJ, USA).

At the bottom of each experimental flask (covered with aluminum foil), a hole was made with a precise diameter matching the LED source (radius of 1.25 cm), corresponding to an area of approximately 4.9 cm^2^ (A = πr^2^). Irradiation was performed at a 90° angle, at a distance of 1 cm, in an anaerobic environment at room temperature (23 °C), in the dark, with no humidity inside the anaerobic chamber. Temperature and humidity were monitored and controlled throughout the experiments.

Irradiance was calculated as the ratio between the optical output power and the irradiated area according to the equation I = P/A, where I is the irradiance, P is the optical output power, and A is the irradiated area. On the basis of an optical output power of 125 mW and an irradiated area of 4.9 cm^2^, the resulting irradiance was approximately 25.5 mW/cm^2^.

Different energy densities were obtained by varying the irradiation time while maintaining a constant irradiance. The relationship between the irradiation time and the delivered energy density is shown in Table 1.

**Table 1:** Relationship between LED Energy Density (J/cm^2^) and Irradiation Time (min)

energy density (J/cm^2^)	irradiation time (min)
8.0	5.20
10.0	6.50
12.0	7.84
14.4	9.40
21.6	14.0

### 2.5 Antimicrobial Photodynamic Inactivation

Initially, preliminary tests were conducted using concentrations of 0.005, 0.010, 0.015, 0.020, 0.025, 0.030, 0.035, 0.045, and 0.050 μg/mL to determine the IC_50_.^26^ As the results did not show significant variations (data not shown), the experiment was adjusted to include concentrations of 0.050, 0.250, 0.500, 0.750, 1.0, 1.5, and 2.0 μg/mL.^26^ At concentrations of 1.0 μg/mL and higher, a reduction in cell viability was observed (data not shown), indicating a cytotoxic effect of DMMB. Consequently, the focus shifted from determining the IC_50_ to assessing the minimum inhibitory concentration.

For the API, DMMB concentrations of 1.0, 1.5, and 2.0 μg/mL were combined with the energy densities described in Table 1. The samples were preincubated in the dark at room temperature (23 °C) for 5 min^26^ before exposure to the light source. Postirradiation, 100 μL aliquots of the suspension were analyzed using a spectrophotometer (SpectraMax 190, Molecular Devices, California, USA) at 600 nm.

All PI procedures were conducted under aseptic conditions inside an anaerobic chamber (Bactron VI, Shellab, Sheldon Manufacturing Inc., USA) without exposure to ambient light, at a controlled temperature of 23 °C, and in a humidity-free environment. The chamber atmosphere consisted of 80% nitrogen (N_2_), 10% carbon dioxide (CO_2_), and 10% hydrogen (H_2_), ensuring optimal experimental conditions.

During each step, 100 μL aliquots of the suspension were added to 96-well plates (Falcon, BD Lab., Franklin Lakes, NJ, USA). Experiments were performed in triplicate for each condition, and the entire experimental set was independently repeated three times to ensure reproducibility. OD_600nm_ was used as a parameter for cell quantification, correlating absorbance with SRB cell concentration.

### 2.6 Statistical Analysis

Multivariate analyses were performed using two-way ANOVA without replication, two-way ANOVA with interaction, and multiple comparisons with the Tukey test, all conducted with GraphPad Prism software version 10.4.1 (San Diego, CA, USA). For all analyses, p values less than 0.05 were considered statistically significant. Results obtained from optical density measurements were converted to estimated cells per milliliters using a proportional relationship based on McFarland turbidity standards.^28^ The equation developed by Edington et al.^29^ was used to calculate the percentage of inhibition achieved in the experiment.

## 3 Results

### 3.1 Determination of DMMB Toxicity

In the groups treated with the photosensitizer DMMB, the results indicated that at concentrations of 0.050, 0.250, 0.500, and 0.750 μg/mL, there was no microbial mortality (Figure 2), with counts of 2.17 × 10^8^ cells/mL, similar to the control. However, at concentrations of 1.0, 1.5, and 2.0 μg/mL, there were reductions in microbial counts to 3.19 × 10^6^, 6.50 × 10^6^, and 1.45 × 10^7^ cells/mL, respectively, compared to the control group, although these differences were not statistically significant (*P* > 0.05).

**Figure 2:**
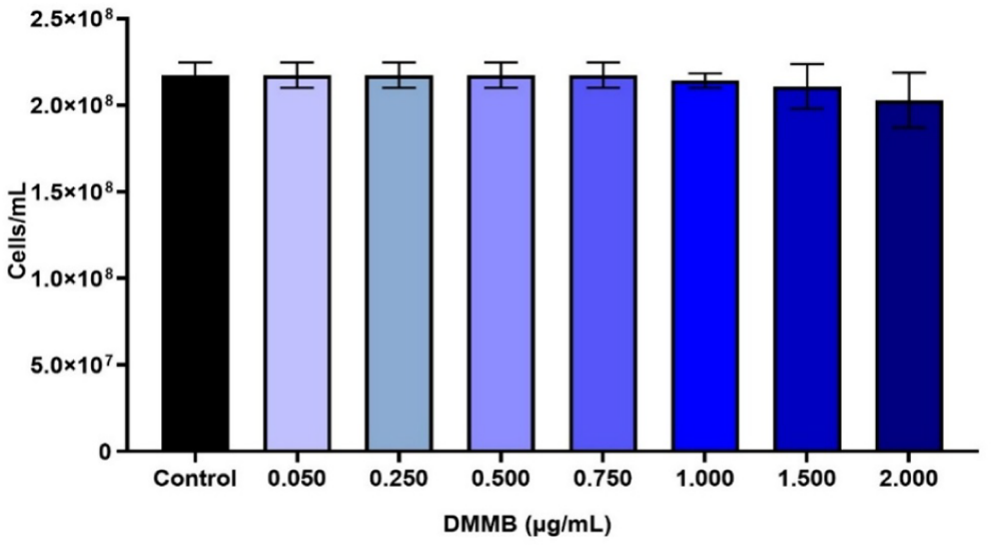
Estimated cell concentrations derived from OD_600nm_ measurements during the DMMB toxicity assay at different concentrations (0.050–2.0 μg/mL).

### 3.2 Evaluation of Different Energy Densities

In all evaluated energy densities (8.0, 10.0, 12.0, 14.4, and 21.6 J/cm^2^), no growth inhibition was observed when compared with the control group (Figure 3). This confirms that the use of an LED light alone, at the tested energy densities, does not exhibit antimicrobial activity.

**Figure 3:**
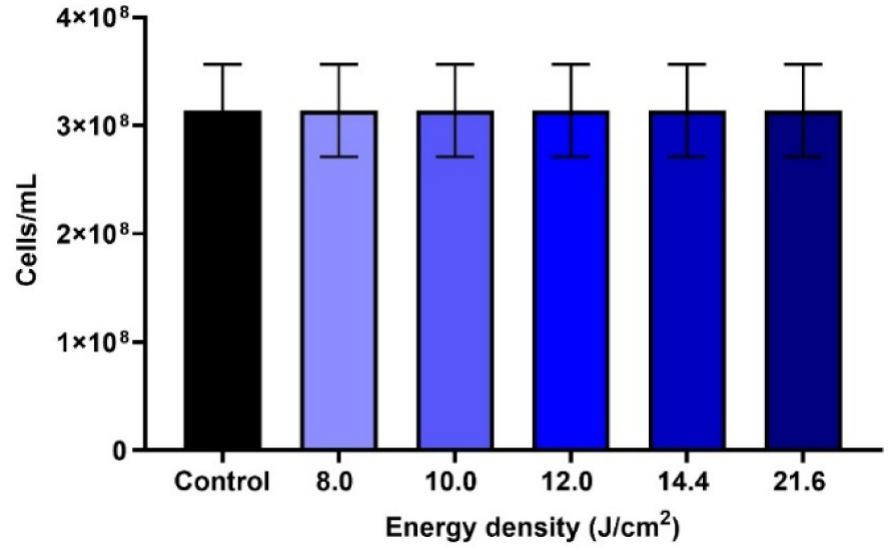
Estimated cell concentrations derived from OD_600nm_ measurements for cultures exposed to LED irradiation at different energy densities (8.0, 10.0, 12.0, 14.4, and 21.6 J/cm^2^).

### 3.3 Antimicrobial Photodynamic Inactivation

The results for the API showed a numerical increase in microbial inhibition with an increasing energy density across the evaluated conditions (Figure 4). However, statistical analysis indicated that variations in the LED energy density did not produce significant differences in microbial mortality at fixed DMMB concentrations.

**Figure 4:**
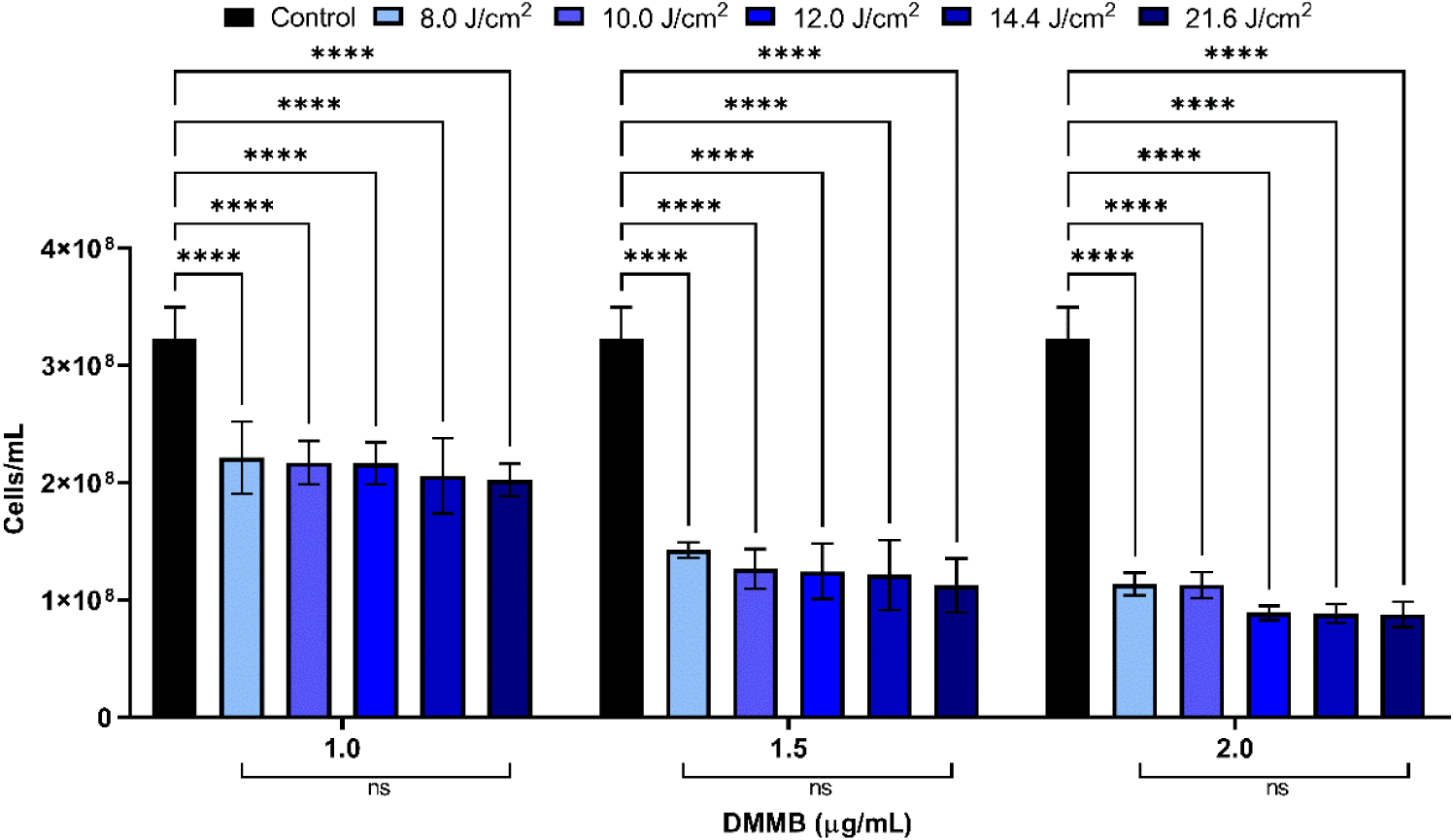
Estimated cell concentrations derived from OD_600nm_ measurements for cultures subjected to antimicrobial photodynamic inactivation under different LED energy densities (8.0, 10.0, 12.0, 14.4, and 21.6 J/cm^2^) combined with DMMB concentrations (1.0, 1.5, and 2.0 μg/mL).

In the intragroup analysis, where DMMB concentration was held constant while varying the five different energy densities, it was observed that there were no significant differences in microbial mortality at any of the three DMMB concentrations used (Figure 3). This means that while increasing the LED energy density numerically increased the percentage of microbial reduction, this increase was not statistically significant. This observation is supported by the two-way ANOVA analysis (Table S1), which evaluated the effects of DMMB concentration, LED energy density, and their interaction. Tukey’s post hoc analysis (simple effects within rows) confirmed significant differences between the control and all LED-treated conditions at each DMMB concentration (*p* < 0.0001), whereas increasing energy density did not result in statistically significant differences within the same photosensitizer concentration (Table S2). Among the comparisons between groups, higher microbial mortality rates were observed with the DMMB concentration of 2.0 μg/mL.

The results obtained from the different concentrations of the photosensitizer DMMB and LED energy densities are presented according to the inhibition measurements and the number of cells per milliliters. From the data analysis, a progressive increase in bacterial growth inhibition was observed primarily with an increasing DMMB concentration, while increases in energy density produced only numerical differences that were not statistically significant.

Inhibition of the SRB consortium increased with both DMMB concentration and energy density. At 8 J/cm,^2^ inhibition ranged from 31.52% (0.16 log) at 1.0 μg/mL to 64.84% (0.45 log) at 2.0 μg/mL, while higher fluence levels (10–21.6 J/cm^2^) further enhanced inhibition, reaching up to 72.86% (0.57 log). This trend demonstrates a positive correlation between energy input and API efficiency, with inhibition plateauing at elevated fluence and photosensitizer levels, suggesting near-saturation of the process. Overall, the combination of higher DMMB concentrations and energy densities resulted in approximately 70% bacterial inhibition (0.52 log), demonstrating the potential of this approach as a proof-of-concept for API of SRB.

## 4 Discussion

### 4.1 Toxicity of DMMB and Antimicrobial Effects of LED Light

As expected, LED light does not exhibit antimicrobial activity. This type of radiation at different energy densities may, in fact, be used to enhance the metabolism of microorganisms in bioprocesses aimed at degrading^30^ or increasing the secretion of target metabolites.^31^

Similarly, the isolated photosensitizer, DMMB, at concentrations of 0.050, 0.250, 0.500, and 0.750 μg/mL did not cause statistically significant cell mortality in the microbial consortium. Although toxicity can be strain-dependent, DMMB at the concentrations tested is generally nontoxic to microorganisms. For pure cultures, toxicity is reported to be absent at concentrations of 0.750 μg/mL,^32^ and up to 100 μg/mL when used with microbial consortia.^33^

It should also be noted that the DMMB preparation used in this study had a reported purity of approximately 80% with the remaining fraction attributed to bound water. The absence of significant antimicrobial activity in the DMMB dark controls suggests that the observed antimicrobial effect primarily resulted from the photoactivation of the photosensitizer. Future studies using higher-purity DMMB preparations could help clarify the potential influence of this dilution by bound water on the API performance.

Additionally, the microbial consortium used in this study was isolated from a harsh environment and had been previously exposed to hydrophilic antimicrobial agents (such as glutaraldehyde), leading to the selection of strains resistant to more toxic compounds than DMMB itself.^34^

Another hypothesis for the low toxicity of DMMB is the cellular nature and protective mechanisms of the bacterial types used. *Desulfovibrio* species, as employed in this study, are known producers of extracellular polymeric substances and biofilms,^35^ which create barriers to the penetration or diffusion of compounds. In this sense, we hypothesize that EPS may bind to DMMB molecules,^36^ hindering their uptake by cells. However, only future research that carefully evaluates the presence or absence of EPS, the specific species producing it, and the uptake of DMMB in the cultures can verify the validity of this statement. Another hypothesis involves alternative mechanisms employed by *Desulfovibrio* *species* to withstand antimicrobial activity, such as the presence of efflux pumps within the membrane that actively export undesirable compounds including antibiotics. These mechanisms may represent an additional barrier limiting DMMB penetration and sufficient interaction with the cells.^37^ As with the previous hypothesis regarding the role of EPS, only future research can confirm or refute this proposition.

### 4.2 API

Despite the ongoing development and application of antimicrobial treatments to control SRB in field applications, several challenges hinder their effectiveness. These challenges include biofilm formation and resistance development.^4^ Biocides used in oil fields face significant hurdles that can impact their performance and operational efficiency. Compatibility with other oilfield chemicals is a major issue; for instance, some biocides can react with bisulfite-based oxygen scavengers,^11^ leading to deactivation. Additionally, certain biocides may form emulsions with oilfield compounds, particularly in the presence of iron sulfide, complicating oil–water separation and affecting production efficiency.

The effectiveness of biocides also depends on their stability under harsh conditions such as varying pH levels, temperatures, and the presence of salts and additives; interactions with these factors can reduce their biocidal properties.^38^ Another challenge is scale formation, where biocides can contribute to issues such as calcium sulfate precipitation, which clogs production systems. Biocides must also penetrate biofilms, which protect microorganisms and make contamination control difficult. Reactions with compounds such as hydrogen sulfide and iron sulfide can further diminish biocide effectiveness. For example, acrolein reacts with H_2_S, while DBNPA and bronopol are deactivated by sulfides and bisulfite-based scavengers.^38^

Finally, the surfactant properties of some biocides, such as quaternary ammonium compounds,^39^ may lead to foaming and interfere with oil–water separation, necessitating careful consideration of their chemical stability and environmental impact. Therefore, reliance on conventional antimicrobial compounds is not sustainable, as it leads to environmental degradation and diminished returns. This highlights the need for new antimicrobial approaches.

Although water-based biocides, including glutaraldehyde and quaternary ammonium compound formulations, are widely used in oilfield operations because of their cost-effectiveness and ease of application, increasing field evidence indicates that sulfate-reducing bacteria can develop tolerance to repeated treatments, particularly under biofilm-forming conditions. Chemical deactivation by sulfides and interactions with oilfield constituents may further reduce biocide efficacy, often necessitating higher dosages or more frequent applications over time.^11^ These limitations motivate the investigation of alternative or complementary antimicrobial strategies, such as API, which operates via a distinct oxidative mechanism and may reduce selective pressure for resistance.

DMMB shows low reactivity with oilfield compounds due to its stable chemical structure. Its central aromatic ring, characteristic of methylene blue, enhances molecular stability,^40^ while the dimethyl substituents further reduce chemical reactivity. Unlike conventional biocides containing reactive groups such as aldehydes, amines, or quaternary ammonium compounds that readily interact with oilfield chemicals,^41^ DMMB lacks these functionalities. Instead, its antimicrobial action depends on the photodynamic generation of cytotoxic species such as singlet oxygen, which selectively targets bacterial membranes owing to DMMB’s positive charge.^20^ This mechanism ensures high antibacterial efficiency while minimizing unwanted reactions with oilfield constituents, contributing to its stability and suitability in complex oilfield environments. Additionally, DMMB’s lipophilic nature may potentially favor interaction with oil–water interfaces,^42^ which could be relevant in petroleum reservoir environments. However, the present experiments were conducted under laboratory conditions and did not include representative reservoir parameters such as multiphase flow, high pressure, elevated temperature, or oil–phase interactions. Therefore, this interpretation requires further validation under more realistic reservoir conditions.

The API-LED groups demonstrated dose-dependent antimicrobial activity. Statistical analysis indicated that photosensitizer concentration was the dominant factor influencing inhibition levels, whereas increases in LED energy density produced only nonsignificant numerical increases in microbial reduction. Previous investigation^22^ has reported the API of sulfate-reducing bacteria using DMMB activated by laser irradiation under anaerobic conditions. In contrast, the present study evaluates the performance of LED-based photoactivation, which represents a technologically more accessible and scalable light source for potential oilfield applications. OD_600nm_ quantifies turbidity rather than viability and is not sensitive to sublethal photodynamic injury. Because OD_600nm_ is dominated by scattering from particulate biomass, dead but structurally preserved cells may still contribute to the signal, whereas reductions in OD_600nm_ more strongly reflect decreases in structurally detectable suspended biomass, including membrane damage, aggregation/settling, and/or lysis. Accordingly, the present data support a dose-dependent reduction in suspended SRB biomass/growth under API conditions rather than a definitive viability-resolved kill endpoint. Future work should incorporate viability-specific assays (e.g., CFU/MPN, LIVE/DEAD fluorescence staining, metabolic assays, or molecular viability approaches) to distinguish lethal from sublethal effects and quantify true inactivation. The results show that using an LED at 10 J/cm^2^ (Table 1) supports the feasibility of an LED as a light source for API, although the observed inhibition levels indicate partial microbial reduction rather than complete SRB control. Also, LEDs offer a lower acquisition cost and a more versatile range and phase compared to other options.^43^

One advantage of this microbial control approach is the short time required for effective API action (16 min) compared to the lengthy duration and recurrence of conventional antimicrobial treatments, such as glutaraldehyde, benzalkonium chloride, and cocodiamine (120 h).^44^ Biocides like chlorine dioxide and quaternary ammonium surfactants can be easily adsorbed by negatively charged particles present in oil wells, reducing their availability for SRB.^42^

On the other hand, the reduction of up to 73% in the microbial population is not very high in terms of microbial control, considering the significant 27% remaining, which can still contribute to the problems caused by SRBs. These results should be interpreted primarily as a proof-of-concept demonstration of API against biocide-resistant SRB consortia rather than as a fully optimized treatment for field-scale microbial control. Incomplete microbial control raises two hypotheses to explain this observation (Figure 5).

**Figure 5:**
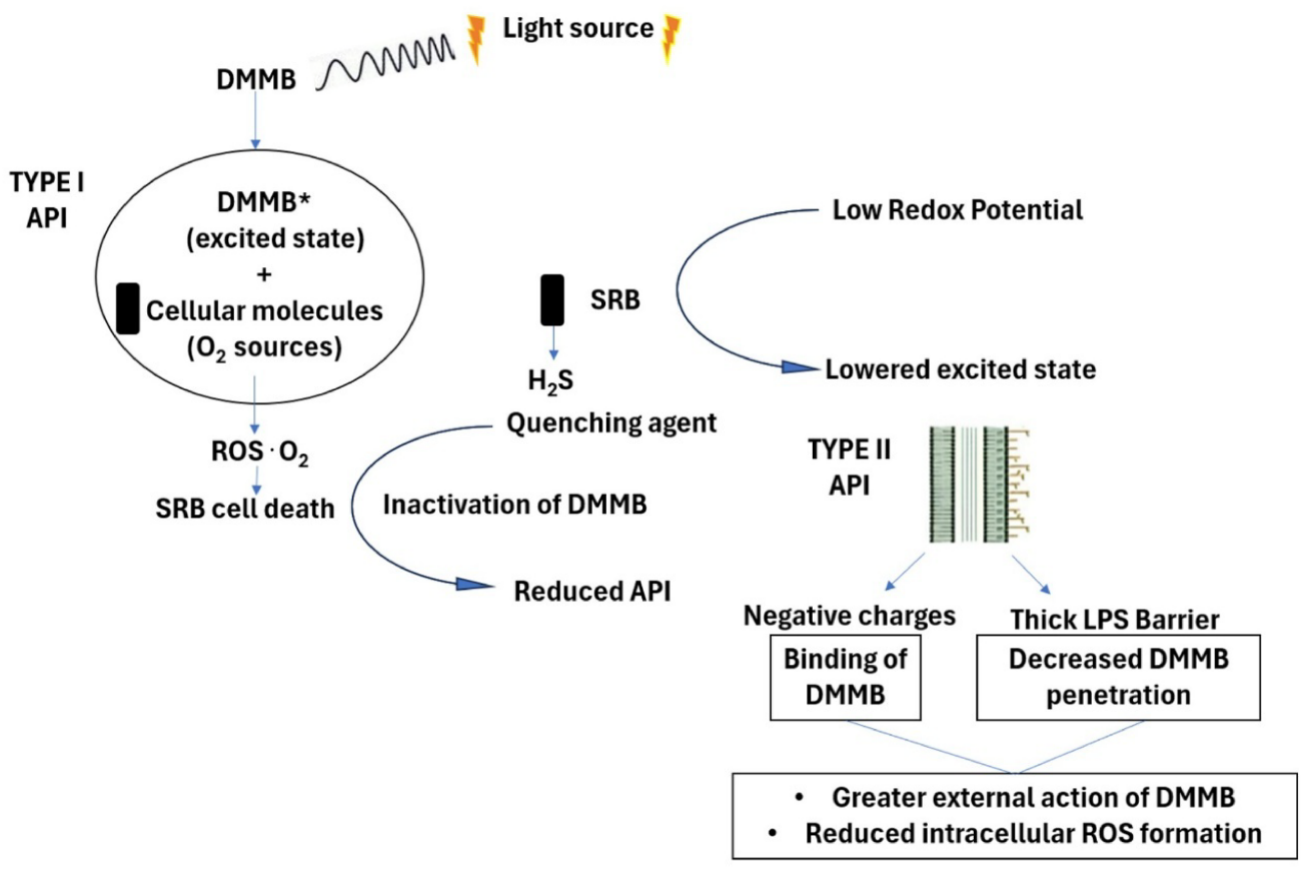
Proposed mechanism of Type I and Type II API-mediated ROS generation and their interactions in SRB inhibition.

The first hypothesis is based on the effects of the conditions generated during SRB growth under the API. Photodynamic activation of photosensitizers can generate reactive oxygen species through two main pathways: type I reactions (producing superoxide, H_2_O_2_, and hydroxyl radicals) and type II reactions (producing singlet oxygen). Since the experiments were conducted under strictly anaerobic conditions, type I photochemical processes are expected to dominate, while type II pathways dependent on molecular oxygen may be significantly limited. The type of ROS produced depends on the photosensitizer structure, redox potential, and surrounding O_2_ concentration.^12,45^ Type II reactions may be the principal mechanism and are greatly influenced by oxygen concentration under aerobic conditions. However, under anaerobic photoactivation, the mechanisms naturally shift to type I reactions, where activated photosensitizers interact with biological substrates on microbial cells (46). However, the present study did not directly measure reactive oxygen species generation, H_2_S concentration, or the redox potential during the photodynamic process. Therefore, the mechanistic interpretation proposed here should be regarded as a plausible hypothesis based on established photodynamic principles rather than as direct experimental evidence.

In the context of singlet oxygen, a quencher is a substance that deactivates this singlet oxygen species (^1^O_2_).^46^ Although ^1^O_2_ is commonly involved in API under aerobic conditions, its contribution may be limited under the strictly anaerobic conditions used in this study. H_2_S is a strong reducing agent and can act directly as a quencher for singlet oxygen by reacting with it, thus reducing the amount of singlet oxygen available to inactivate microorganisms.^46^ This reaction typically results in the oxidation of H_2_S and the reduction of singlet oxygen to its ground state O_2_. This quenching reduces the reactive oxygen species (ROS) available for causing cellular damage during the API process. It has been demonstrated that the supplementation of the singlet oxygen quencher l-tryptophan significantly decreased the antibacterial activity of the methylene blue photosensitizer on *Desulfovibrio vulgaris* ATCC29579 and *Desulfovibrio desulfuricans* ATCC14563 under aerobic conditions of LED photoactivation, with less effect under anaerobic photoactivation, which supports the proposed argument.^46^ Although this quenching mechanism has been reported in previous studies, the interaction between H_2_S and singlet oxygen was not directly quantified in the present work.

Furthermore, the generation of H_2_S by SRB also lowers the redox potential of the medium, making it more reductive.^42^ Photosensitizers like DMMB rely on specific redox reactions to transition from their ground state to an excited state upon light activation. A low redox potential may reduce the efficiency of these redox reactions, leading to less effective excitation of the photosensitizer. This shift could potentially impact DMMB’s ability to transition into its active state upon light exposure, resulting in reduced production of singlet oxygen. While this hypothesis is plausible, the present study did not experimentally measure redox potential or photosensitizer photochemistry under these conditions. Further experiments are needed to determine how redox potential and H_2_S influence DMMB-mediated API.

Considering the strictly anaerobic experimental conditions employed, the antimicrobial effects observed during API are more likely associated with type I photochemical reactions involving electron transfer and radical formation rather than classical type II singlet oxygen pathways.

The second hypothesis is based on the chemical nature of DMMB and the morphological and functional characteristics of the SRB. DMMB is highly lipophilic, which may initially be advantageous for penetration into the cells of the SRB consortia. *Desulfovibrio* species have a complex outer membrane and lipopolysaccharide (LPS) bilayer^47^ that significantly hinders the entry of hydrophilic antimicrobial compounds. However, LPS present on SRB outer membranes confers a pronounced negative charge,^48^ which might facilitate interaction with DMMB through electrostatic forces. Additionally, LPS creates a complex barrier that prevents positively charged molecules from penetrating the cell and interacting with DNA, limiting the effectiveness of the photosensitizer to the outer membrane (as seen with positively charged antimicrobial peptides in Gram-negative bacteria like *E. coli*).^49^ Similarly, when *Desulfovibrio vulgaris* was exposed to a positively charged intercalating dye (propidium monoazide), incomplete penetration and DNA intercalation were reported even after cell death by thermal inactivation.^50^ This may suggest incomplete DMMB penetration, insufficient to generate intracellular singlet oxygen for effective photodynamic damage,^32^ thus limiting the effectiveness of the API. Nevertheless, photosensitizer uptake or intracellular localization was not experimentally quantified in this study, and the proposed limitation of intracellular penetration should therefore be interpreted as a mechanistic hypothesis that requires future validation.

DMMB API has been employed in *Candida albicans* cultures, achieving a 99.991% log reduction,^32^ while its rhamnolipid nanoencapsulated negatively charged form at the same concentration achieved a 99.9% log reduction.^32^ *Candida albicans* has a cell wall composed mainly of glucans, mannans, and chitin^51^ and lacks an outer membrane, resulting in higher permeability to cationic compounds. Conversely, the negatively charged nature of its outer membrane tends to decrease the affinity for negatively charged molecules. Similarly, *Enterococcus faecalis* (Gram-positive) subjected to DMMB API yielded a 99.999998% log reduction,^21^ while Gram-positive *Staphylococcus aureus* achieved a 99.97% log reduction.^27^ These bacteria have a less complex cell wall and lack the outer LPS barrier found in *Desulfovibrio* strains but possess a peptidoglycan layer. Although this layer is positively charged because of its amino acid content, the presence of teichoic acids and carboxylate teichuronic acids adds overall negative charges to the cell wall. This contributes to the nonselective permeability of most Gram-positive bacteria to cationic compounds.^52^

When comparing these results with methylene blue-based API for controlling SRB, similar outcomes were observed in the inactivation of *Desulfovibrio vulgaris* ATCC and *Desulfovibrio desulfuricans* ATCC biofilm cultures using 100 and 500 ng/mL of methylene blue API with LED treatment.^46^ It is important to note that this study utilized a real-world microbial consortium from an oilfield that had been treated with biocides, resulting in enhanced antimicrobial mechanisms suited to such an environment. Furthermore, the study employed *Brucella* Blood Agar enriched with hemin and vitamin K to grow the SRB strains.^46^ When *Desulfovibrio* species are grown on this medium, they may produce less H_2_S compared to liquid media such as Postgate C, as substrate and metabolic product diffusion is limited in solid media. This limitation could reduce the bacterial metabolic activity and resistance development to methylene blue. Additionally, blood components may influence bacterial metabolism, potentially decreasing oxidative stress and thereby reducing the resistance mechanism for the employed API treatment.

In contrast, Postgate C medium is specifically designed to support SRB growth and robust H_2_S production by providing sulfate as an electron acceptor. Furthermore, in addition to the very high photosensitizer concentration (≈100 μg/mL), the study demonstrated that aerobic conditions had a key effect on the API process, suggesting enhanced generation of ROS, such as singlet oxygen, which improved the API efficacy in pure planktonic cultures. However, using such high photosensitizer concentrations increases both the cost of the technology and its environmental impact, while oxygen injection poses operational risks due to the high flammability of hydrocarbons.^46^

In liquid media, bacteria have better access to nutrients^53^ and electron donors/acceptors, enhancing their metabolic activity and H_2_S production. This may justify the similar 73% log reduction observed when using 750 ng/mL DMMB photosensitizer on such resistant SRB consortia. On the basis of these results, approximately 27% of the initial cell load remained, suggesting a potential selective pressure on the microbial community that could lead to the development of more resistant microorganisms with the sequential use of API combined with DMMB. This hypothesis should be explored in future studies.

A recent study conducted by dos Santos et al.^27^ employed LASER light combined with the dye DMMB to treat microbial SRB consortia, showing promising results with 71% inhibition when using 2.0 μg/mL of DMMB, comparable to those observed in the present study. However, this study highlighted the superiority of using LED light, which offers practical, economic, and environmental advantages, making it a more efficient and accessible alternative.

LEDs stand out as significantly more cost-effective than lasers, both in terms of acquisition and maintenance. Their simpler technology eliminates the need for sophisticated systems, making LEDs more viable for resource-limited laboratories and expanding access to this technique.^45^ Additionally, LEDs provide greater safety by emitting diffuse light, minimizing the risk of damage to adjacent tissues or surfaces. In contrast, LASERs, due to their high intensity and focused nature, require strict controls to avoid side effects, which may limit their applicability in certain contexts.^12^

LEDs not only directly compete with lasers but also offer greater practical feasibility. Moreover, LEDs have the advantage of covering larger areas uniformly, making them ideal for large-scale applications such as surface treatments and industrial processes. LASERs, on the other hand, are more limited in such applications due to their highly directional nature.

Another important advantage of LEDs is their sustainability. With greater energy efficiency and an extended lifespan, LEDs reduce long-term operational costs and have a lower environmental impact. Their potential for technological adaptation is also a key differentiator, as ongoing advancements in LED technology promise to increase their intensity and efficiency, further narrowing the performance gap.^54^

Although the results do not achieve a high reduction for practical SRB control, they lay the groundwork for exploring APIs with biocide-resistant consortia isolated from oilfields and grown in chemical media that support SRB activity and H_2_S production. However, these laboratory conditions do not fully reproduce reservoir environments, and further studies under representative operational parameters will be required to assess field applicability. Additionally, the mechanistic interpretations discussed in this study are based on indirect observations and literature-supported reasoning, as direct measurements of ROS production, H_2_S concentration, redox potential, and photosensitizer uptake were beyond the scope of the present work. This highlights the need for further investigations to confirm the proposed hypotheses regarding (I) the quenching activity of H_2_S on singlet oxygen and (II) the morpho-functional specificities of *Desulfovibrio* strains. Consequently, the present findings should be interpreted as an initial demonstration of the potential of LED-mediated API for SRB management with further optimization required to achieve inhibition levels compatible with practical field applications.

In this study, DMMB was employed as a model photosensitizer, not with the intent of proposing its direct deployment in oilfield operations, but to generate mechanistic insight into the API of biocide-resistant sulfate-reducing bacteria under realistic growth conditions. DMMB was selected due to its well-characterized photophysical properties and established use as a reference compound in API research, enabling systematic evaluation of how photosensitizer concentration, LED energy density, microbial physiology, and environmental factors such as H_2_S production influence treatment efficacy. From a toxicity and cost perspective, API using DMMB differs fundamentally from conventional water-based oilfield biocides such as DBNPA and glutaraldehyde–quaternary ammonium (Glut–Quat) combinations. While DBNPA and Glut–Quat formulations are widely applied due to their rapid action, water solubility, and relatively low initial cost, their repeated use has been associated with acute aquatic toxicity, occupational health risks, chemical deactivation by sulfides or organic matter, biofilm-mediated tolerance, and increasing dosage requirements over time,^41^ which can offset their apparent economic advantages. In contrast, DMMB was applied here at low concentrations and activated only under controlled illumination, where antimicrobial activity is mediated catalytically via light-induced generation of reactive oxygen species rather than continuous chemical toxicity. This mode of action limits uncontrolled toxicity in the absence of light and reduces reliance on bulk chemical dosing. Although aromatic photosensitizers such as DMMB may face regulatory constraints for large-scale environmental use, the mechanistic insights gained in this work provide a benchmark for the rational design and evaluation of greener and more regulatory-compatible alternatives, including naturally derived photosensitizers, immobilized or polymer-bound systems, and nanoformulations with reduced environmental release. A rigorous comparison of ecotoxicity, life-cycle cost, and regulatory compliance between API and conventional biocides such as DBNPA or Glut–Quat would require dedicated ecotoxicological and techno-economic analyses and is therefore beyond the scope of the present study, but represents an important direction for future research.

## 5 Conclusion

This study explores the effects of API using the photosensitizer DMMB activated by LED lights, applied to a biocide-resistant souring microbial consortium isolated from an oilfield. The following conclusions can be drawn:

(A) A consortium of sulfate-reducing bacteria isolated from fully operational oilfields represents a more suitable microbial model for API, as it includes more resistant strains that pose a greater challenge for the API.
(B) The complex relationship between the physicochemical properties of the photosensitizer and the morpho-functional characteristics of the consortium cells may influence the site of action of API. The detailed mechanisms should be explored in future research to evaluate hypotheses regarding cell membrane properties, transport mechanisms, and the potential presence of microbial metabolites interacting with the DMMB.
(C) H_2_S produced by SRB metabolism may act as a quencher, potentially reducing the overall effectiveness of the type II API reactions. This hypothesis should be investigated through additional experiments.

The hypotheses proposed, while plausible, require validation through future experimental research. The groundwork laid here provides guidelines for investigating the use of DMMB as a photosensitizer in API treatment of SRB in oilfield environments, addressing unanswered questions in the current state of the art of API with DMMB and LED for SRB control. Future work should also focus on investigating strategies to enhance the efficacy of API in such microbial consortia at the bench scale. These strategies may include the use of nanoencapsulated photosensitizers, eco-friendly metal–photosensitizer hybrids, and the evaluation of micro- to low-oxygen exposure during the API process.

## Supplementary Material



## Data Availability

The data underlying this study are available in the published article.

## Notes

Table S1: Two-way ANOVA table for the data shown in Figure 4; Table S2: Tukey’s multiple-comparisons test (simple effects within rows) following two-way ANOVA for the data shown in Figure 4 (PDF)
