# Supplementary material for: 1,9-Dimethyl-methylene Blue-Based Antimicrobial Photodynamic Inactivation: Sulfate-Reducing Bacteria Models Isolated from Oilfield Wastewater
Source: ACS Omega. 2026 Apr 20;11(17):25014–24. doi: 10.1021/acsomega.5c09000 (PMC13150645; doi:10.1021/acsomega.5c09000)
Supplement: Supplementary file 1 [file ao5c09000_si_001.pdf]

1,9-Dimethyl-Methylene Blue-Based Antimicrobial Photodynamic Inactivation: Sulfate-Reducing Bacteria Models Isolated from Oilfield Wastewater

Hesrom Fernandes Serra Moura[1], Igor Carvalho Fontes Sampaio[2,3*], Gustavo Vital dos Santos[1], Anna Paula Lima Teixeira da Silva[1], Pedro Jorge Louro Crugeira[4], Wellington Luis Reis Costa[1], Antonio Luiz Barbosa Pinheiro[1], Paulo Fernando de Almeida[2]

[1]Center of Biophotonics, School of Dentistry, Federal University of Bahia – UFBA, 62, Araújo Pinho Ave, Canela, Salvador, BA, CEP: 40110 -150, Brazil.

[2]Laboratory of Biotechnology and Ecology of Microorganisms, Institute of Health Sciences, Federal University of Bahia - UFBA, Av. Reitor Miguel Calmon, S/N, 40110-100, Salvador, BA, Brazil.

[3]Biotransformation and Organic Biocatalysis Research Group, Department of Exact Sciences, Universidade Estadual de Santa Cruz, 45654-370 Ilhéus, Brazil.

[4]CIMO, LA SusTEC, Instituto Politécnico de Bragança, Campus de Santa Apolónia, 5300-253, Bragança, Portugal.

[*]Corresponding author.
Laboratory of Biotechnology and Ecology of Microorganisms, Institute of Health Sciences, Federal University of Bahia - UFBA, Av. Reitor Miguel Calmon, S/N, 40110-100, Salvador, BA, Brazil.

Biotransformation and Organic Biocatalysis Research Group, Department of Exact Sciences, Universidade Estadual de Santa Cruz, 45654-370 Ilhéus, Brazil.

E-mail address: igorsampaio@outlook.com

SUPPLEMENTARY TABLES

Table S1. Two-way ANOVA table for the data shown in Figure 4

Source	SS (Type III)	DF	MS	F (DFn, DFd)	P value
Interaction	4.531e+016	10	4.531e+015	F (10, 85) = 10.42	p < 0.0001
Row Factor	1.631e+017	2	8.154e+016	F (2, 85) = 187.6	p < 0.0001
Column Factor	5.741e+017	5	1.148e+017	F (5, 85) = 264.1	p < 0.0001
Residual	3.695e+016	85	4.347e+014		

Table S2. Tukey's multiple-comparisons test (simple effects within rows) following two-way ANOVA for the data shown in Figure 4

Row level	Comparison	Mean difference	95% CI	Adjusted p
1	Control vs 8.0 J/cm²	1.02×10^8	6.71×10^7 to 1.36×10^8	<0.0001
	Control vs 10.0 J/cm²	1.06×10^8	7.32×10^7 to 1.39×10^8	<0.0001
	Control vs 12.0 J/cm²	1.07×10^8	7.51×10^7 to 1.38×10^8	<0.0001
	Control vs 14.4 J/cm²	1.17×10^8	8.00×10^7 to 1.54×10^8	<0.0001
	Control vs 21.6 J/cm²	1.21×10^8	8.61×10^7 to 1.55×10^8	<0.0001
1.5	Control vs 8.0 J/cm²	1.80×10^8	1.39×10^8 to 2.21×10^8	<0.0001
	Control vs 10.0 J/cm²	1.96×10^8	1.65×10^8 to 2.28×10^8	<0.0001
	Control vs 12.0 J/cm²	1.99×10^8	1.58×10^8 to 2.40×10^8	<0.0001
	Control vs 14.4 J/cm²	2.02×10^8	1.67×10^8 to 2.36×10^8	<0.0001
	Control vs 21.6 J/cm²	2.11×10^8	1.76×10^8 to 2.45×10^8	<0.0001
2	Control vs 8.0 J/cm²	2.09×10^8	1.79×10^8 to 2.40×10^8	<0.0001
	Control vs 10.0 J/cm²	2.10×10^8	1.80×10^8 to 2.41×10^8	<0.0001
	Control vs 12.0 J/cm²	2.34×10^8	1.99×10^8 to 2.69×10^8	<0.0001
	Control vs 14.4 J/cm²	2.35×10^8	1.98×10^8 to 2.72×10^8	<0.0001
	Control vs 21.6 J/cm²	2.35×10^8	1.98×10^8 to 2.73×10^8	<0.0001
